# Impact of Incremental Peritoneal Dialysis on Cardiometabolic Parameters

**DOI:** 10.7759/cureus.83107

**Published:** 2025-04-28

**Authors:** Osasuyi Iyasere, Aasiya Mahomed, Lara Caracciolo

**Affiliations:** 1 Cardiovascular Sciences, John Walls Renal Unit, University Hospitals of Leicester National Health Service (NHS) Trust, Leicester, GBR; 2 Department of Cardiovascular Sciences, University of Leicester, Leicester, GBR; 3 College of Medicine, Leicester Medical School, University of Leicester, Leicester, GBR

**Keywords:** cardiovascular, glucose, incremental, metabolic, peritoneal dialysis

## Abstract

Background

Glucose is the predominant osmotic agent of peritoneal dialysis (PD) fluid. The systemic exposure to glucose experienced by people receiving PD has been linked to insulin resistance and cardiovascular morbidity. Incremental PD has been associated with a lower exposure to glucose, but little is known about the impact on measures of insulin resistance. We hypothesised that patients on incremental PD would have a lower glucose-related cardiometabolic burden. This study aimed to compare trends in cardiometabolic parameters, including serum glucose, haemoglobin A1c (HbA1c), cholesterol, body mass index (BMI), and body weight, between patients receiving incremental and full-dose peritoneal dialysis.

Materials and methods

This was a retrospective study with a two-year follow-up period. Adults who had received PD between 2015 and 2022 were included. The eligibility criteria included a minimum PD duration of 12 months and a 24-hour urine volume of at least 500 mL at the onset of PD. Demographic and clinical data, including PD prescriptions, were collated. Cardiometabolic parameters, including dry weight, BMI, HbA1c, cholesterol and serum glucose levels, were collected as outcome measures at six-monthly intervals.

Eligible participants were categorised into incremental PD and full-dose PD groups based on prespecified criteria. The PD prescriptions were used to estimate cumulative peritoneal glucose exposure. Generalised linear mixed methods analysis was used to evaluate the effect of incremental PD on the trend in outcome measures.

Results

A total of 117 (incremental = 46; full dose = 71) patients were included in the study. There was no significant difference in baseline demographic or clinical parameters, except for HbA1c, which was lower in the incremental cohort (5.3 [5.2 to 6.6]) compared to the full dose cohort (6.6 [6.0 to 7.5], p = 0.04). The estimated cumulative glucose exposure was substantially lower in the incremental cohort (65kg at 24 months) compared to the full-dose group (105kg at 24 months, p < 0.001). After adjusting baseline characteristics, higher serum glucose was associated with full-dose PD compared to incremental PD (coefficient = 0.17 [0.00 to 0.35], p = 0.05), although there was no difference in the trend over time. There was also no significant difference in trends for BMI, dry weight, cholesterol or HbA1c.

Conclusion

In this retrospective cohort study, cumulative dialysate glucose exposure and serum glucose levels were significantly lower with incremental PD compared to full-dose PD. However, there was no significant difference in cardiometabolic trends over time. Prospective studies will help establish the potential benefits of incremental therapies on cardiometabolic parameters and cardiovascular risk in PD patients.

## Introduction

Peritoneal dialysis (PD) is a home-based form of kidney replacement therapy (KRT) for people with end-stage kidney disease (ESKD). It involves the exchange of dialysate in the peritoneal cavity. PD offers flexibility and independence to those for whom the impact of dialysis on lifestyle is a key determinant of their KRT choice. These benefits have informed initiatives to increase the utilisation of home therapies in people requiring dialysis [1].

Most people receiving PD have some residual kidney function at the onset of treatment [2]. Residual kidney function has been associated with favourable clinical outcomes in the PD population. These outcomes include lower mortality risk [3] and lower peritonitis rates [4]. Several studies have reported a strong association between mortality and residual kidney function in patients receiving PD. A re-analysis of the CANUSA study, involving 601 patients, found that every 0.5 ml/min/year increase in residual glomerular filtration rate (GFR) was associated with a 12% lower risk of death [3]. The International Society of Peritoneal Dialysis (ISPD) has published guidelines on PD prescriptions, which emphasise patient-centred, goal-directed therapy that recognises and preserves residual kidney function [5].

Incremental peritoneal dialysis is a treatment strategy that considers the presence of residual kidney function. It involves the initiation of PD at a lower dose, with a clear intention to increase the dialysis dose as residual kidney function declines and/or uraemic symptoms appear [2].

There are several proposed benefits to incremental PD, which include the potential to preserve residual kidney function. Incremental dialysis was associated with a slower decline in residual kidney function in a meta-analysis of 22 cohort studies involving HD and PD patients [6]. In contrast, there was no difference in the rate of decline in residual kidney function in a randomised trial comparing three with four manual exchanges in patients receiving continuous ambulatory PD [7]. Incremental PD is associated with a reduction in the number of dialysis connections, time spent receiving dialysis treatment and dialysate exposure. These potential benefits may improve quality of life and reduce PD peritonitis rates [8].

Glucose is the predominant osmotic agent in the peritoneal dialysate. The exposure to glucose in PD is thought to lead to local peritoneal effects and systemic metabolic effects [8]. An estimated 88 grams of glucose are absorbed daily during PD [9]. This additional caloric load may exacerbate adverse effects of insulin resistance, such as hyperglycaemia, hyperlipidaemia, hyperinsulinaemia, and obesity. These cardiometabolic parameters have been linked to increased cardiovascular risk. Incremental PD is associated with reduced peritoneal exposure to glucose, compared to full-dose PD [7]. However, it is unclear whether incremental PD has an impact on measures of insulin resistance and consequent cardiovascular risk.

We hypothesised that patients on incremental PD would have a lower glucose-related cardiometabolic burden. This study aimed to compare trends in cardiometabolic parameters, including serum glucose, HbA1c, cholesterol, BMI, and body weight, between patients receiving incremental and full-dose peritoneal dialysis using a two-year retrospective cohort design. This article was previously presented as a meeting abstract at the UK Kidney Week 2024 conference on June 12, 2024.

## Materials and methods

This was a retrospective cohort study with two years of follow-up. It was conducted at University Hospitals of Leicester NHS Trust. The John Walls Renal Unit (JWRU) research database was interrogated to identify eligible patients who had received PD up until 2022. This was followed by approval from the Data Release Group at JWRU. Ethical approval for the JWRU research database was obtained from the Yorkshire and the Humber - Leeds East Research Ethics Committee (REC reference number - 22/YH/0104).

The inclusion criteria were as follows: age of 18 years or above, a minimum PD duration of 12 months, and a 24-hour urine volume of at least 500 mL at the onset of PD. Those who had received haemodialysis for at least three months or had received a previous kidney transplant or who did not meet the inclusion criteria were excluded. 

Demographic and clinical data were collected for the eligible participants at baseline. These included age at the onset of PD, ethnicity, sex, diabetes status and socioeconomic status as defined by the index of deprivation. The PD prescriptions and 24-hour urine volume were collected at baseline. PD prescriptions were also collected during follow-up to capture changes in the dialysis dose over time. Data relating to dry weight, BMI, HbA1c, glucose and cholesterol levels were collected as outcome measures. These were collected six monthly, for up to two years. The baseline timepoint was defined as any time within one month after the onset of PD. The extracted data were independently cross-checked by two researchers.

The identified cohort was categorised into incremental and full-dose groups, based on prespecified criteria at the onset of PD. The criteria for incremental continuous ambulatory peritoneal dialysis (CAPD) included any of the following: less than four manual exchanges/day, less than seven days per week, and an exchange volume of less than two litres per exchange. The criteria for incremental automated peritoneal dialysis (APD) included any of the following: less than seven nights a week; absence of a daytime exchange; and a total dialysate volume of less than eight litres per therapy [2]. Those who did not meet these criteria were considered to be receiving a full dose of PD.

The baseline characteristics between the incremental and full-dose PD groups were compared using descriptive statistics. Continuous data were expressed as the mean or median, depending on whether the data was normally distributed or not. Normally distributed data were compared using the T-test, while non-parametric data were compared using the Mann-Whitney test. Categorical data were expressed as percentages and compared using Fisher's exact test. The dialysis prescriptions were used to estimate cumulative glucose exposure over two years. The calculations considered changes in dialysis prescriptions during follow-up and assumed full adherence to prescriptions. A worked example is shown in the Appendix.

Generalised linear mixed model analysis was used to compare trends in the outcome measures between the incremental and full-dose PD groups. Age, sex, ethnicity, diabetes status and index of deprivation were considered as potential predictors or covariates. Results with a p-value less than or equal to 0.05 were considered to be statistically significant. The predicted values from each model were plotted over time to depict the trends in outcome measures. Missing data were deemed to be missing at random. Data imputation methods were therefore not utilised, as mixed model analysis is able to handle missing data.

## Results

A total of 117 patients met the eligibility criteria and were included in the study. There were 46 patients in the incremental PD cohort. Seventy-one patients were included in the full-dose PD cohort. Eleven patients in the incremental cohort had their dialysis dose increased during the study period, four of whom reached full dose. The median age for the entire cohort was 60 years (interquartile range = 50 to 71). A total of 47% were female, while 76% were of white ethnicity. A total of 20.5% of the cohort were diabetic, and the majority were on APD (91%). 

Table 1 shows the demographic and clinical characteristics at baseline. There was no significant difference in the demographic characteristics between the incremental and full-dose PD groups. The full-dose PD cohort had a significantly higher proportion of patients with APD compared to the incremental PD cohorts. There was no difference in the use of non-glucose peritoneal dialysate (extraneal or nutrineal). The full-dose PD group also had statistically higher HbA1c levels at baseline and a higher volume of peritoneal dialysate used per week. There was no difference in the other clinical characteristics at baseline.

**Table 1:** Baseline demographic and clinical characteristics APD: Automated peritoneal dialysis; BMI: Body mass index; IQR: Interquartile range

Variable	Incremental PD	Full-dose PD	P-value
Age (years)	63 (53 to 71)	59 (49 to 71)	0.38
Female sex (%)	47.9	46.5	0.80
Ethnicity (%)			0.77
Asian	14.1	10.1	
Black	4.2	5.8	
White	77.1	75.4	
Other	0.0	1.4	
Unknown	4.2	7.2	
Diabetes (%)	27.1	15.9	0.17
BMI (%)	25.1 (22.7 to 26.5)	26.2 (23 to 28.8)	0.31
Cholesterol (mmol/L) (median and IQR)	4.2 (4.3 to 5.2)	3.9 (3.1 to 4.8)	0.61
Hba1c (%) (median and IQR)	5.3 (5.2 to 6.6)	6.6 (6.0 to 7.35)	0.04
Index of deprivation (median and IQR)	7 (4 to 9)	6 (3 to 7)	0.07
24-hour urine volume (litres) (median and IQR)	1.67 (0.71 to 1.71)	1.49 (0.75 to 1.54)	0.94
Non-glucose peritoneal dialysate use (%)	8.7%	7%	0.73
APD (%)	81.2%	98.2%	< 0.01
Volume of peritoneal dialysate used per week (litres) (median and IQR)	52.5 (49 to 56)	77 (64.6 to 84)	< 0.01

**Figure 1:**
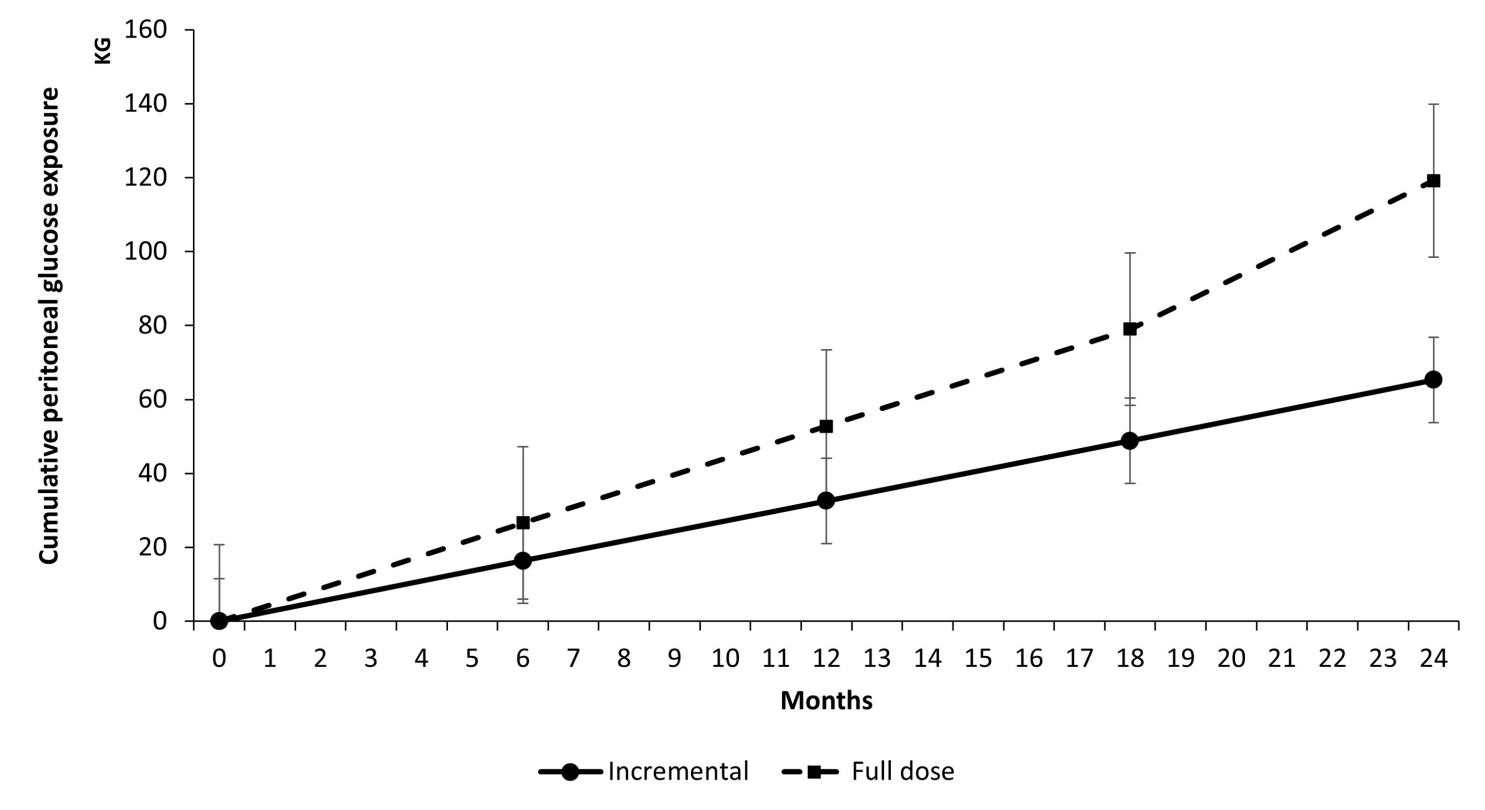
Cumulative peritoneal glucose exposure over time KG: Kilograms

The cumulative PD glucose exposure increased in both cohorts during follow-up. It increased more rapidly in the full-dose PD cohort compared to the incremental PD cohort, as shown in Figure 1. The mean cumulative PD glucose exposure at two years was 65 ± 2.6 kg in the incremental cohort and 105 ± 2.7 kg in the full-dose cohort (p < 0.0001).

Outcome measures

Body Mass Index and Weight

**Figure 2:**
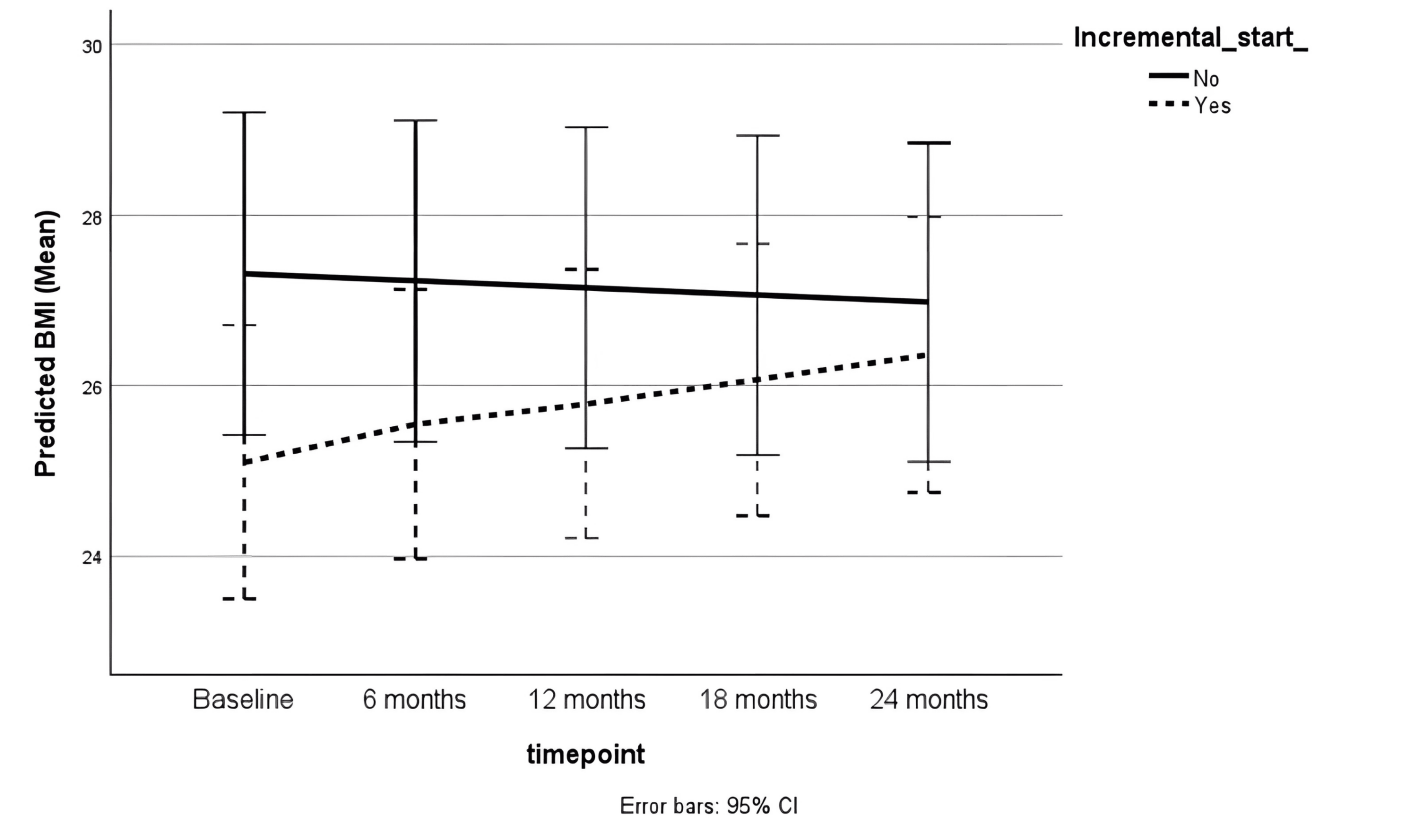
BMI trend during follow-up BMI: Body mass index; CI: Confidence interval

**Figure 3:**
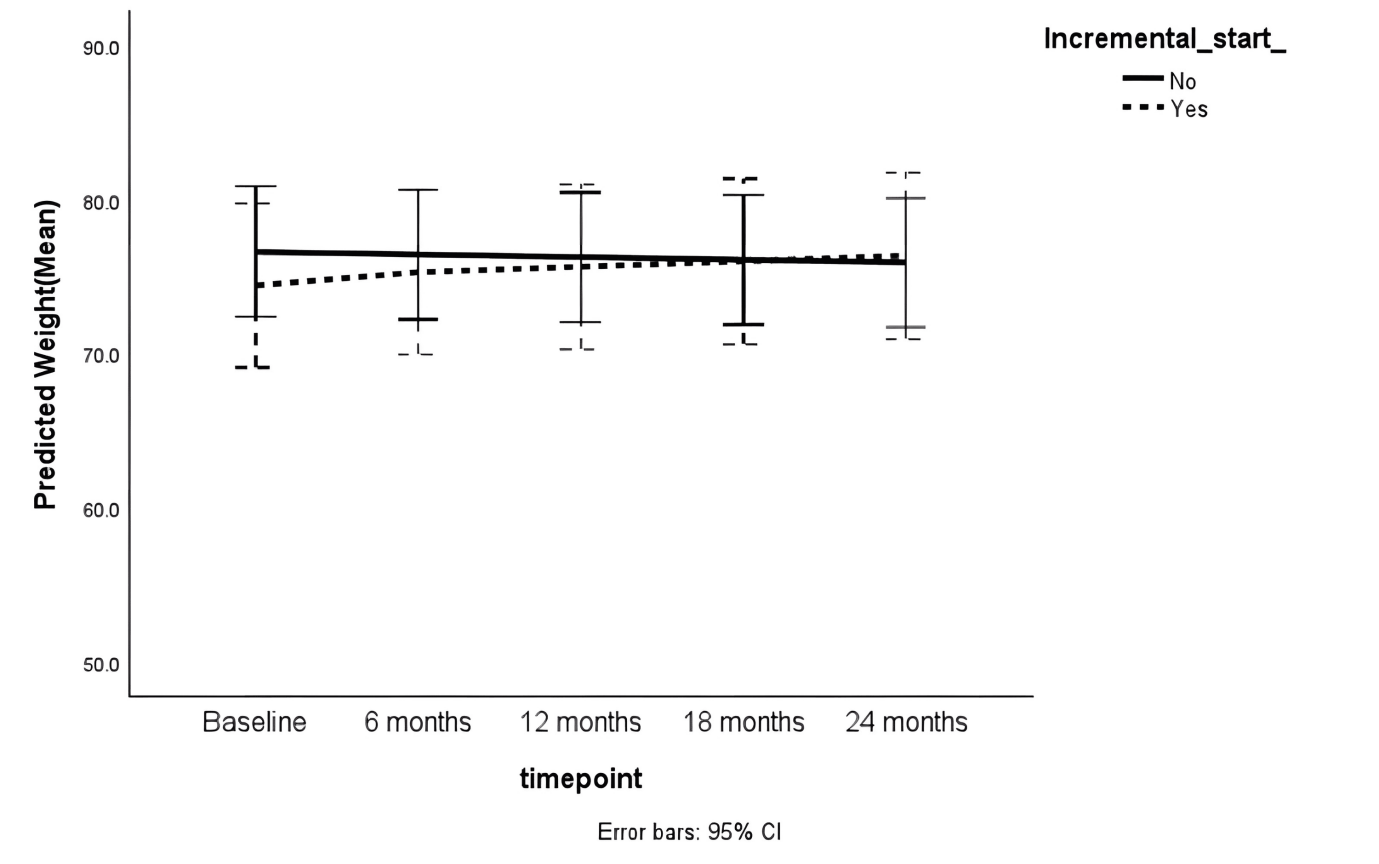
Trend in dry weight (kg) during follow-up CI: Confidence interval

There was a trend towards an increase in BMI during the follow-up period in the adjusted generalised linear mixed model (coefficient = 0.11 [-0.01 to 0.23], p = 0.06). There was no statistically significant difference in the BMI trends between incremental and full-dose PD groups (Table 2). Female sex was significantly associated with lower dry weight (coefficient = -0.11 [-0.20 to -0.02], p = 0.01). However, there was no significant change over time and no difference between the incremental and full-dose PD cohorts (Table 3). Figures 2, 3 show the trends in BMI and dry weight, respectively.

**Table 2:** Generalised linear mixed model: outcome – body mass index

Model Term	Coefficient	P-value	95% Confidence Interval	
			Lower	Upper
Intercept	3.311	< 0.01	2.947	3.676
Age	-0.001	0.67	-0.005	0.003
Female	0.023	0.69	-0.091	0.136
Male (reference)	0			
Ethnicity: Asian	0.045	0.76	-0.239	0.328
Ethnicity: Black	0.134	0.52	-0.541	0.273
Ethnicity: Other	-0.004	0.99	-0.439	0.431
Ethnicity: White	-0.104	0.39	-0.340	0.132
Ethnicity: Unknown (reference)	0			
Non-diabetic	0.033	0.67	-0.118	0.184
IMD decile=1	-0.014	0.92	-0.295	0.267
IMD decile=2	-0.001	0.99	-0.213	0.210
IMD decile=3	-0.133	0.26	-0.365	0.100
IMD decile=4	0.279	0.01	0.079	0.478
IMD decile=5	0.096	0.34	-0.101	0.292
IMD decile=6	-0.233	0.04	-0.451	-0.016
IMD decile=7	-0.009	0.92	-0.179	0.162
IMD decile=8	-0.040	0.68	-0.233	0.152
IMD decile=9	0.034	0.46	-0.058	0.126
IMD decile=10 (reference)	0			
Time	0.011	0.07	-0.001	0.023
Full-dose PD	0.075	0.23	-0.047	0.198
Incremental PD (reference)	0			
Full-dose PD*Time	-0.014	0.06	-0.029	0.001
Incremental PD*Time (reference)	0			

**Table 3:** Generalised linear mixed model: outcome – dry weight (kg)

Model Term	Coefficient	P-value	95% Confidence Interval	
			Lower	Upper
Intercept	4.184	<0.01	3.834	4.535
Ethnicity: Asian	0.047	0.69	-0.183	0.277
Ethnicity: Black	0.077	0.57	-0.190	0.343
Ethnicity: Other	0.039	0.87	-0.418	0.496
Ethnicity: White	0.192	0.06	-0.005	0.389
Ethnicity: Unknown (reference)	0			
Female	-0.112	0.01	-0.200	-0.024
Male (reference)	0			
Incremental PD	-0.028	0.56	-0.121	0.066
Full-dose PD (reference)	0			
Time	0.005	0.27	-0.004	0.013
Incremental PD*Time	0.007	0.20	-0.004	0.018
Full-dose PD*Time (reference)	0			

Serum Cholesterol, Glucose and HbA1c

**Figure 4:**
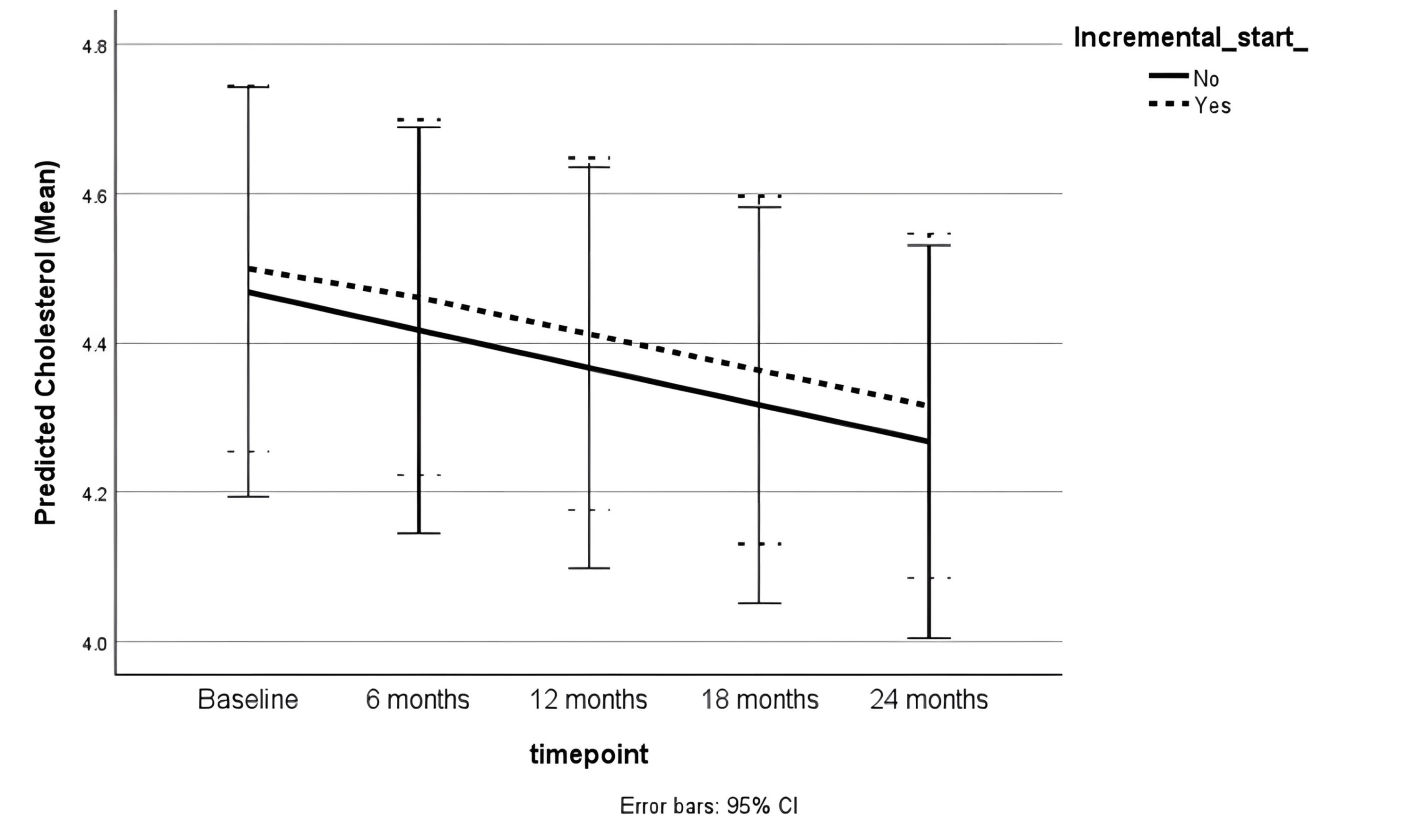
Trend in serum cholesterol (mmol/L) during follow-up CI: Confidence interval

The female sex was associated with higher serum cholesterol (coefficient = 0.12 (0.04 to 0.20), p = 0.01) in the adjusted analysis. Serum cholesterol did not change significantly over time and was not associated with incremental or full-dose PD (Table 4). Figure 4 shows the trends in serum cholesterol in both groups.

**Table 4:** Generalised linear mixed model: outcome – serum cholesterol (mmol/L) PD: Peritoneal dialysis

Model Term	Coefficient	P-value	95% Confidence Interval	
			Lower	Upper
Intercept	1.444	<0.01	1.353	1.534
Female	0.119	0.01	0.035	0.203
Male (reference)	0			
Time	-0.011	0.16	-0.026	0.004
Incremental PD	0.015	0.79	-0.092	0.122
Full-dose PD (reference)	0			
Incremental PD*Time	0.0004	0.97	-0.020	0.021
Full-dose PD*Time	0			

**Figure 5:**
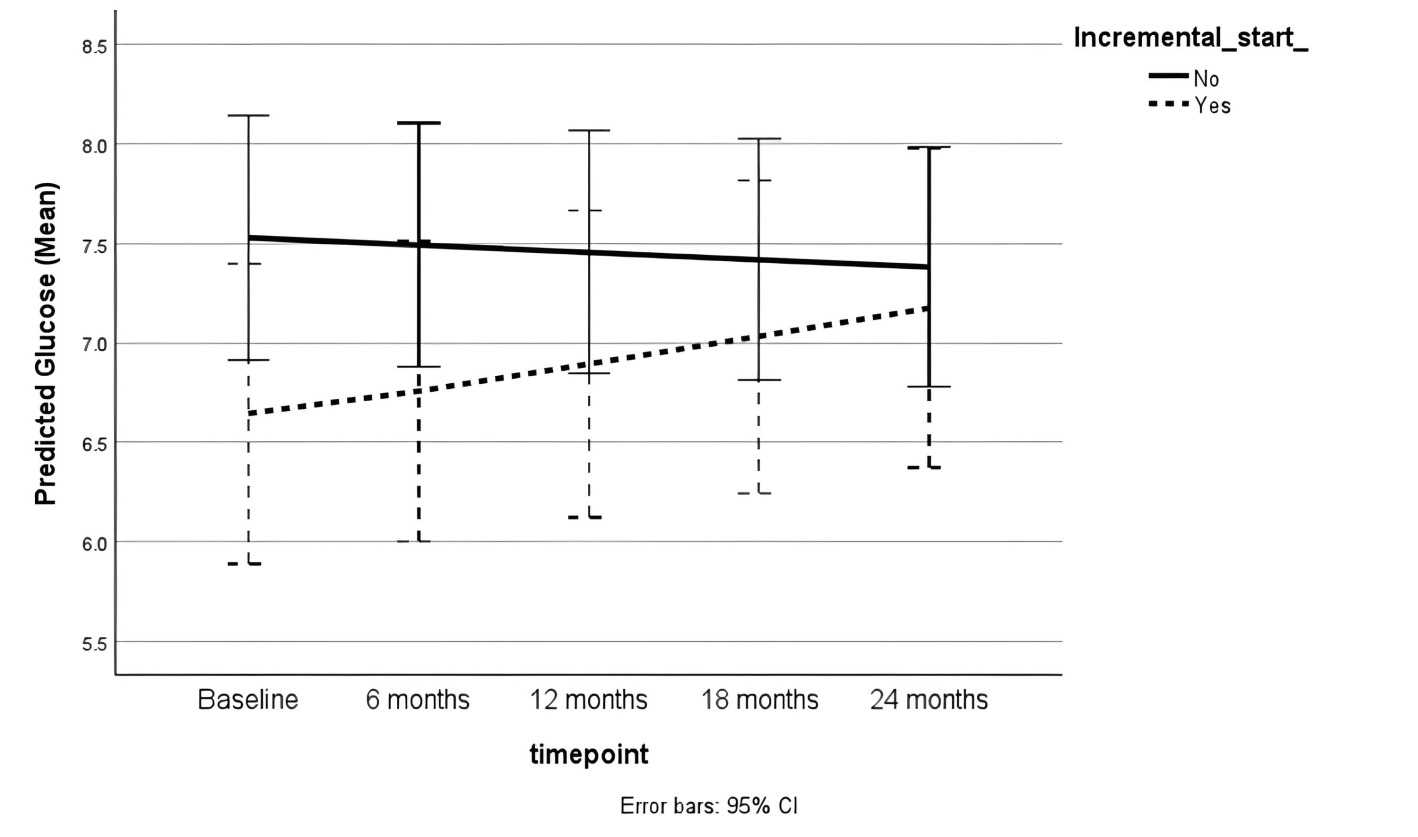
Trend in serum glucose (mmol/L) during follow-up CI: Confidence interval

Non-diabetic patients had lower serum glucose levels compared to diabetic participants. Higher serum glucose was associated with full-dose PD compared to incremental PD, as shown in Table 5 (coefficient = 0.17 [0.00 to 0.35, p = 0.05]). However, there was no significant difference in trend over time (Figure 5). In the non-diabetic subgroup, the incremental PD cohort had a lower proportion of participants with random serum glucose levels greater than 11.1 mmol/L during follow-up, compared to those on full-dose PD (11.8% vs. 31%, p = 0.04).

**Table 5:** Generalised linear mixed model: outcome – serum glucose (mmol/L) PD: Peritoneal dialysis

Model Term	Coefficient	P-value	95% Confidence Interval	
			Lower	Upper
Intercept	2.311	0.01	1.973	2.648
Ethnicity: Asian	-0.241	0.18	-0.592	0.110
Ethnicity: Black	-0.457	0.03	-0.875	-0.038
Ethnicity: Other	0.049	0.90	-0.680	0.778
Ethnicity: White	-0.387	0.01	-0.680	-0.094
Ethnicity: Unknown (reference)	0			
Diabetic	0.197	0.03	0.019	0.375
Non-diabetic (reference)	0			
Time	0.020	0.18	-0.009	0.049
Incremental PD	0.174	0.05	-0.349	0.002
Full-dose PD (reference)	0			
Incremental PD*Time	0.025	0.20	-0.013	0.063
Full-dose PD*Time (reference)	0			

**Figure 6:**
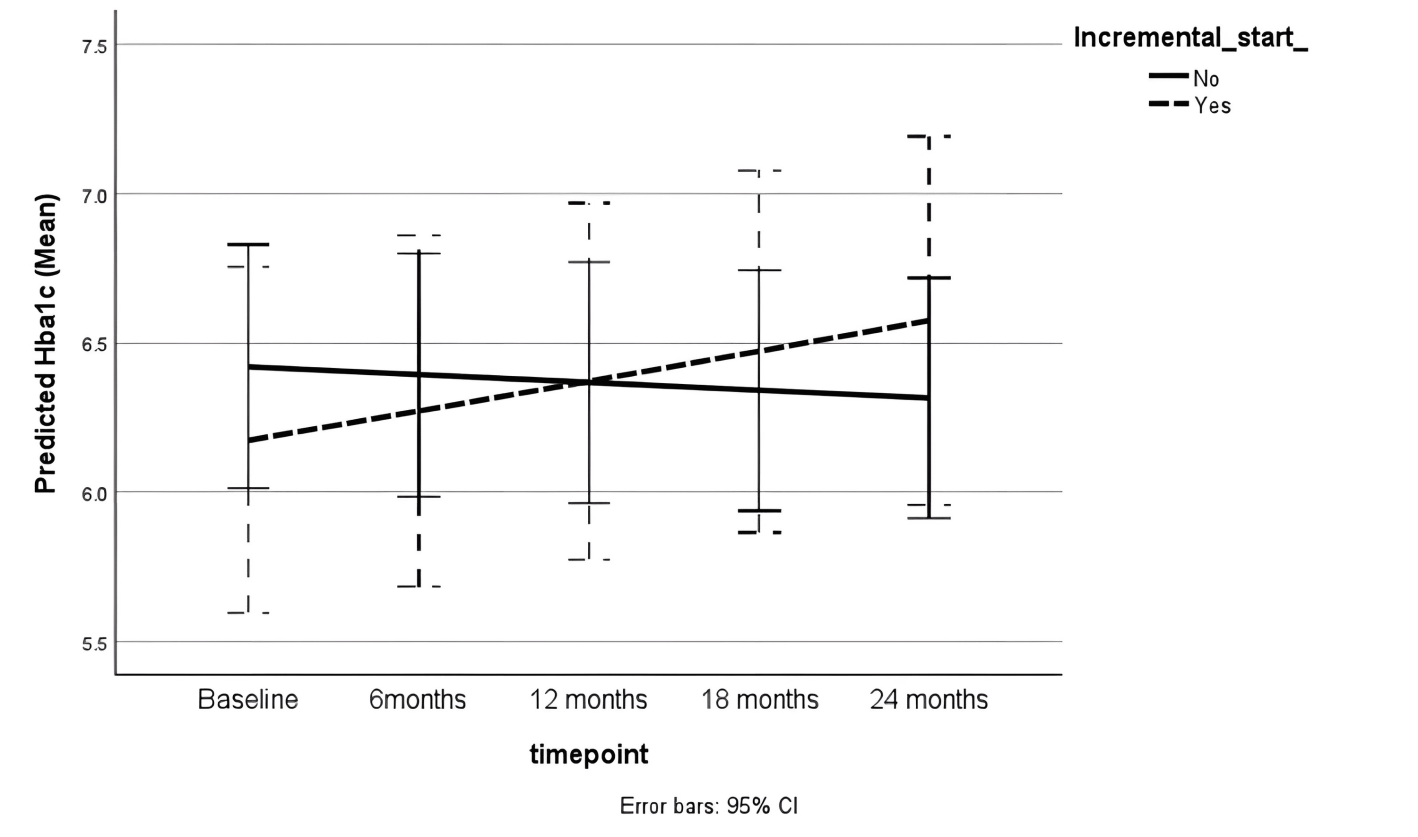
Trend in HbA1c during follow-up CI: Confidence interval

In the subgroup of diabetic patients, there was no significant difference in HbA1c trends between the incremental or full-dose PD groups (Figure 6) after adjusting for age, sex, ethnicity and socioeconomic status (Table 6).

**Table 6:** Generalised linear mixed model: outcome – HbA1c (%) IMD decile: 1 is redundant

Model Term	Coefficient	P-value	95% Confidence Interval	
			Lower	Upper
Intercept	2.181	0.01	1.649	2.712
Age	-0.003	0.19	-0.009	0.002
Ethnicity: Asian	-0.131	0.48	-0.494	0.233
Ethnicity: Black	0.030	0.89	-0.409	0.468
Ethnicity: White	-0.306	0.10	-0.674	0.061
Ethnicity: Unknown (reference)	0			
Female	0.005	0.93	-0.111	0.121
Male (reference)	0			
IMD decile=1				
IMD decile=2	-0.160	0.18	-0.393	0.073
IMD decile=3	0.326	<0.01	0.120	0.531
IMD decile=4	0.167	0.12	-0.045	0.380
IMD decile=5	0.080	0.67	-0.292	0.452
IMD decile=6	0.296	0.02	0.060	0.532
IMD decile=7	0.127	0.23	-0.081	0.334
IMD decile=8	0.065	0.50	-0.126	0.256
IMD decile=9	0.039	0.73	-0.179	0.257
IMD decile=10 (reference)	0			
Incremental PD	0.008	0.92	-0.139	0.155
Full-dose PD (reference)	0			
Time	0.016	0.15	-0.006	0.037
Incremental PD*Time	0.020	0.23	-0.013	0.052
Full-dose PD*Time (reference)	0			

## Discussion

It is hypothesised that an incremental approach may ameliorate the metabolic effects associated with PD. In this single-centre retrospective cohort study, patients receiving incremental PD were exposed to a lower cumulative amount of peritoneal glucose compared to those on full-dose PD. While serum glucose levels were generally higher with the full-dose PD cohort compared to incremental PD during follow-up, cardiometabolic trends did not differ between the two groups.

Lower glucose exposure from peritoneal dialysate has previously been associated with incremental PD. In a randomised controlled trial of incident patients receiving CAPD, peritoneal glucose exposure was lower with three daily PD exchanges compared to four daily PD exchanges (100g/day versus 127g/day, p <0.001) [7]. In our study, cumulative glucose exposure was lower in the incremental group. By contrast, our cohort predominantly consisted of patients receiving APD. There is a high correlation between the glucose concentration in peritoneal dialysate and the amount of glucose absorbed in CAPD patients [10]. In a more recent pilot study of patients with APD, while there was a degree of correlation between the amount of glucose absorbed and peritoneal dialysate concentration, the amount absorbed was lower than reported in CAPD patients. This is partially thought to be due to shorter dwells associated with APD [9].

There are conflicting reports on the association between peritoneal glucose and metabolic parameters. In a cross-sectional study of 51 non-diabetic PD patients, peritoneal glucose was not associated with BMI, relative fat-free mass or Homeostatic Model Assessment for Insulin Resistance (HOMA-IR) [11]. Increased peritoneal glucose exposure was also not associated with an increase in body fat in a cohort study of 139 anuric PD patients [12]. In contrast, the prevalence of metabolic syndrome increased after the onset of PD, in a cohort study of 195 non-diabetic patients. Peritoneal glucose exposure was also independently associated with the development of metabolic syndrome [13]. Despite the difference in cumulative glucose exposure in our study, there was no difference in BMI or weight between the full dose and incremental PD groups.

Serum glucose levels are reportedly associated with peritoneal dialysate glucose exposure. In the GLOBAL Fluid cohort study of non-diabetic PD patients, random serum glucose levels increased with daily dialysate glucose (β = -0.0002, 95% CI -0.0004 to -0.00006). 3.7% of incident PD patients were found to have glucose levels consistent with undiagnosed diabetes [14]. In our study, incident patients receiving full-dose PD had higher cumulative PD glucose exposure with higher serum glucose levels during follow-up, compared with the incremental PD. The full-dose PD cohort also had a higher proportion of non-diabetic patients with random glucose levels reaching diabetic thresholds during follow-up. It is not known whether these patients were diagnosed with diabetes based on associated symptoms, as that data was not available. Nevertheless, hyperglycaemia is associated with reduced vascular compliance and atherosclerotic plaque progression, which are linked with increased cardiovascular risk [15]. 

This study has other important limitations. The observational nature of the study limits the results to associative rather than causal relationships. The study included participants with a minimum PD duration of 12 months. The cohort was therefore highly selected, with outcomes that may not be generalisable. Due to the retrospective nature of the study, the estimated peritoneal glucose exposure was based on an assumption of full adherence to PD prescriptions. However, a systematic review has reported that non-adherence, defined as performing less than 90% of prescribed exchanges, affects up to 40% of PD patients [16]. It has been postulated that incremental PD may be associated with improved adherence due to a lower burden of treatment. It has also been speculated that patients receiving incremental PD may be reluctant to adhere to a subsequent increase in the prescription [2]. It is possible that the study outcomes were affected by actual differences in patient adherence between patients on incremental and full-dose PD.

There were also limitations in the outcome measures that could be evaluated. Data on other measures of adiposity, such as waist-hip ratio or triceps skinfold thickness, were not available. Moreover, the data on lipid profiles was restricted to total cholesterol levels, whereas uraemic and diabetic dyslipidaemia are characterised by high triglycerides and low HDL cholesterol [17]. These parameters may therefore be more responsive to changes in glycaemic load. Data on potential modifiers of the study outcomes, such as dietary intake, physical activity and exogenous insulin use, were not available. Peritoneal solute transfer rate (PSTR) and residual kidney function may be associated with markers of metabolic syndrome [18,19]. However, robust data on PSTR and changes in residual kidney function during follow-up were also not available. They therefore could not be considered in the mixed model analysis. Despite these limitations, this is the first study to evaluate the impact of incremental PD on cardiometabolic parameters, to our knowledge.

## Conclusions

In this retrospective cohort study, incremental PD was associated with lower cumulative peritoneal glucose exposure and lower serum glucose levels, compared to full-dose PD during follow-up. It was also associated with a lower proportion of non-diabetic patients with serum glucose levels reaching diabetic thresholds during follow-up. There were no significant differences in other cardiometabolic parameters. Randomised clinical trials that compare cardiometabolic trends and cardiovascular outcomes between the two PD prescription strategies would improve the quality of evidence on the potential cardiovascular benefits of incremental therapies.

## Appendices

Worked example for peritoneal glucose exposure

CAPD prescription - 3 exchanges (2 Litres of 1.36% Dianeal x 2 exchanges; 2 Litres of 2.27 Dianeal x 1 exchange) for 6 days per week; increased to 7 days per week after 18 months.

Weekly peritoneal glucose exposure per exchange = Dialysate glucose concentration (g/L) x exchange volume x number of days.

Weekly peritoneal glucose exposure per 1.36% Dianeal bag for 6 days (A)- 13.6g/L x 2 L x 6 = 163.2g

Weekly peritoneal glucose exposure per 1.36% Dianeal bag for 7 days (B)- 13.6g/L x 2L x 7 = 190.4g

Weekly peritoneal glucose exposure per 2.27% Dianeal bag for 6 days (C) - 22.7g/L x2L x 6 = 272.4g

Weekly peritoneal glucose exposure per 2.27% Dianeal bag for 7 days (D) - 22.7g/L x2L x7 = 317.8g

Weekly peritoneal glucose exposure (6-day prescription) (E) = 2A + 1C = 435.6g

Weekly peritoneal glucose exposure (7-day prescription) (F) = 2B +1D = 698.6g

6 months = 26 weeks

12 months = 52 weeks

18 months = 78 weeks

Cumulative peritoneal glucose exposure after 2 years = (E x 78 weeks) + (F x 26 weeks) = (435.6 x 78) + (698.6 x26) = 52140.4 g or 52.1kg
